# Molecular mechanisms of STIM/Orai communication

**DOI:** 10.1152/ajpcell.00007.2016

**Published:** 2016-04-15

**Authors:** Isabella Derler, Isaac Jardin, Christoph Romanin

**Affiliations:** ^1^Institute of Biophysics, Johannes Kepler University of Linz, Linz, Austria; and; ^2^Department of Physiology, University of Extremadura, Cáceres, Spain

**Keywords:** CRAC channel, STIM1, Orai, STIM-Orai interaction, Orai gating

## Abstract

Ca^2+^ entry into the cell via store-operated Ca^2+^ release-activated Ca^2+^ (CRAC) channels triggers diverse signaling cascades that affect cellular processes like cell growth, gene regulation, secretion, and cell death. These store-operated Ca^2+^ channels open after depletion of intracellular Ca^2+^ stores, and their main features are fully reconstituted by the two molecular key players: the stromal interaction molecule (STIM) and Orai. STIM represents an endoplasmic reticulum-located Ca^2+^ sensor, while Orai forms a highly Ca^2+^-selective ion channel in the plasma membrane. Functional as well as mutagenesis studies together with structural insights about STIM and Orai proteins provide a molecular picture of the interplay of these two key players in the CRAC signaling cascade. This review focuses on the main experimental advances in the understanding of the STIM1-Orai choreography, thereby establishing a portrait of key mechanistic steps in the CRAC channel signaling cascade. The focus is on the activation of the STIM proteins, the subsequent coupling of STIM1 to Orai1, and the consequent structural rearrangements that gate the Orai channels into the open state to allow Ca^2+^ permeation into the cell.

ca^2+^ is a major intracellular messenger in eukaryotic cells. Changes in intracellular Ca^2+^ concentration are required for many physiological processes ranging from fast events like exocytosis to long-term processes like proliferation (13, 14, 74). Store-operated calcium channels (SOCs) constitute the main Ca^2+^ entry pathway into the cell (1, 11, 12, 16, 34, 36, 42, 43, 50, 62, 77, 78, 118, 119, 125, 139, 143, 196) and occur in almost all cell types (3, 11, 12, 16, 144).

While the discovery of store-operated Ca^2+^ ion channels has a long history since the 1970s and 1980s, the molecular players that choreograph store-operated Ca^2+^ entry have only been investigated within the past decade (5, 46, 78, 84, 125, 134, 137, 170, 185). Already in the early 1980s, the idea arose that cells contain cytosolic Ca^2+^ stores, which release Ca^2+^ after stimulation by certain physiological agonists (58, 108, 134, 163). This depletion of internal stores has been supposed to enable external Ca^2+^ to enter the cell, thus triggering diverse signaling cascades that cause, among other processes, secretion, gene transcription, or cell proliferation (19, 47). The most prominent store-operated Ca^2+^ entry pathway is represented by the Ca^2+^ release-activated Ca^2+^ (CRAC) channel (118). Thereby, Ca^2+^ store depletion from the endoplasmic reticulum (ER) is initiated via binding of inositol 1,4,5-trisphosphate (IP_3_) to its respective receptor. Upon a drop in ER Ca^2+^ levels through IP_3_ receptor-mediated Ca^2+^ release, CRAC channels activate to refill these stores and initiate responses by a drastic increase in cytosolic Ca^2+^ levels (46).

Two proteins, i.e., stromal interaction molecule 1 (STIM1) and Orai1 (also called CRACM1), were revealed only in 2005–6, via function-based genetic screen by systematic RNA interference, as the key components fully reconstituting CRAC channel function (19, 46, 84, 103, 123, 137, 152, 185). In addition, the severe combined immune deficiency (SCID) syndrome, which is accompanied by a defect in CRAC channel function, cleared the way to the discovery of the Orai1 channel protein and its mutant Orai1 R91W, occurring in SCID patients (46).

STIM1 represents the ER-located Ca^2+^-sensing stromal interaction molecule (84, 137, 153, 186), which senses the ER Ca^2+^ content by its luminal EF-hand in the NH_2_ terminus. The Orai channel is the plasma membrane (PM) pore-forming subunit of the CRAC channel (46, 84, 128, 170, 178, 181, 185, 186), which is made up of four transmembrane (TM) domains flanked by cytosolic NH_2_ and COOH termini (47, 60, 170) and forms highly Ca^2+^-selective pores located in the PM. After Ca^2+^ store depletion, STIM1 multimerizes and redistributes into discrete puncta close to the PM (9, 18, 22, 121, 178). Subsequently, STIM1 couples to and stimulates Orai1, initiating pronounced CRAC currents (110, 111). It has been demonstrated in vitro that these two proteins are indispensable and sufficient to fully restore store-operated Ca^2+^ currents (191).

The discovery of these two proteins has enabled us to obtain a basic understanding of the course of events starting with the release of Ca^2+^ from the ER and finally culminating in Ca^2+^ entry from the extracellular side. A further milestone in the characterization of STIM and Orai proteins was reached in 2012–13 by crystallization of cytosolic fragments of STIM1 (161, 162, 180, 190) and the full-length Orai channel of *Drosophila melanogaster* (dOrai) (63). Moreover, the structure of a complex of STIM1 and Orai1 COOH-terminal fragments has been resolved by NMR (161). All these structures have provided further resolution of intra- and intermolecular interactions and represent a basis to derive potential conformational changes from the closed to the active state. This review focuses on the molecular mechanisms of STIM1/Orai communication.

## STIM and Orai Proteins

## 

### STIM proteins.

The STIM protein family includes two members, STIM1 and STIM2 (150), which are both expressed in the ER (84, 95, 151, 186). A lower amount has also been detected in the PM, which is, however, not necessarily required for CRAC channel activation (2, 18, 95). The two isoforms are closely related and share ∼61% sequence identity (18). Among metazoans, from *Caenorhabditis elegans* and *Drosophila* to *Homo sapiens*, STIM proteins are highly conserved (27).

**Fig. 1.**
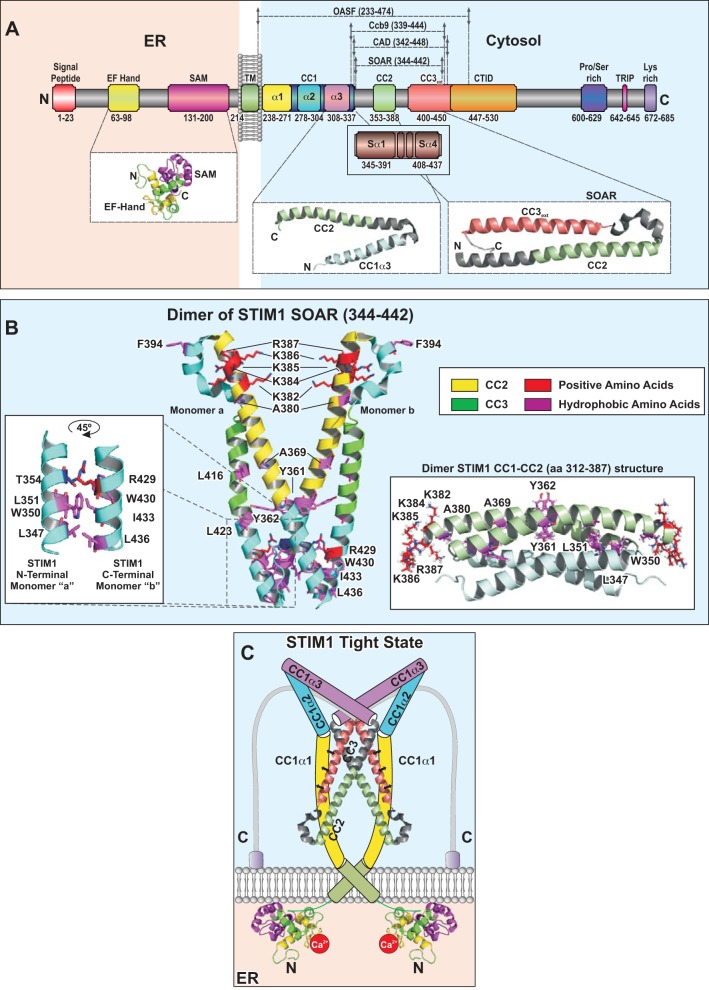
Stromal interaction molecule 1 (STIM1). *A*: scheme depicts full-length, human STIM1 with regions essential for the STIM1/Orai1 signaling cascade. *Insets*: structures of the EF-sterile-α-motif (SAM) domain, the STIM1 coiled-coil domain (CC)1_α3_-CC2 fragment as well as the STIM1 SOAR (344–442) fragment. ER, endoplasmic reticulum; OASF, Orai1 activating STIM1 fragment; CTID, COOH-terminal inhibitory domain; TRIP, Thr-Arg-Ile-Pro sequence. *B*: crystallographic structure of a STIM1 SOAR (344–442) dimer, exhibiting a V-shaped form, includes CC2 and CC3. Amino acids involved in dimer interaction as well as those mediating coupling to Orai1 (positively charged residues) are highlighted. *Left inset*: magnified view of interacting residues between the NH_2_-terminal portion of SOAR monomer “a” and the COOH-terminal portion of SOAR monomer “b.” *Right inset*: NMR structure of a STIM1 CC1_α3_-CC2 dimer. The STIM1 CC1_α3_-CC2 monomers kink between the 2 coiled-coil domains and form dimers via coupling in an antiparallel manner. *C*: hypothetical model of STIM1 in the resting state.

STIM proteins (Fig. 1) are single TM proteins with an NH_2_-terminal portion including the Ca^2+^-sensing domain localized within the ER lumen and a long cytosolic strand, which couples to Orai channels in the PM. The STIM NH_2_ and COOH termini are separated by an ∼20-amino acid (aa) TM region (18, 84, 137) (Fig. 1*A*). The NH_2_-terminal portion is well conserved from worms and flies to humans, while the COOH-terminal portions are rather divergent among different species (20). The variety of STIM proteins is further extended by diverse splice variants like STIM1L (30) and a STIM2 splice variant (STIM2β, STIM2.1) (104, 136).

The Ca^2+^-sensing domain is formed by the EF-hand and sterile-α-motif (SAM) domain (Fig. 1*A*), which display distinct properties in STIM1 and STIM2 (162, 190). Upon store depletion, the STIM EF-hand loses Ca^2+^ and alters its communication with the SAM domain (190), which represents the initial step in the CRAC signaling cascade. This signal is transmitted to the cytosolic portion and finally transforms STIM1 into its active conformation.

The STIM1 TM domain (aa 214–233; Fig. 1*A*) has been recently reported to undergo conformational changes from a potential closed state to the open state (92), thus contributing to the transmission of the stimulatory signal from the luminal side to the COOH terminus. This TM domain contains three small and highly flexible glycines at positions 223, 225, and 226 (56, 92, 177) potentially involved in close helix-helix packing or the introduction of kinks (38).

The STIM1 cytosolic domain (Fig. 1*A*) includes the essential site(s) for coupling to Orai1 (63, 64, 111). It consists of three conserved cytosolic coiled-coil (CC) domains (aa: C1 233–343, CC2 353–388, CC3_ext_ 400–450) followed by the CRAC modulatory domain (aa475–483), the COOH-terminal inhibitory domain (CTID, aa447–530), the serine/proline (Ser/Pro)-rich region, a Thr-Arg-Ile-Pro (TRIP) sequence, and the lysine-rich region at the very end of the COOH terminus (56, 57). On the basis of structural predictions, the 110-residue-long CC1 can be subdivided into three α-helical regions (aa: CC1_α1_ 238–271, CC1_α2_ 278–304, CC1_α3_ 308–337) (150, 161, 180). A recent crystal structure of a STIM1 COOH-terminal fragment (SOAR aa344–442), representing the minimal Orai activating fragment and including CC2 and CC3, has revealed four separate regions (Fig. 1*A*), i.e., Sα1 (aa345–391), Sα2 (aa393–398), Sα3 (aa400–403), and Sα4 (aa409–437) (180). STIM1 and STIM2 display significant differences in their COOH-terminal portion, while the CC domains are well conserved (56). The SOAR/CAD/Ccb9 (71, 121, 183) fragment (Fig. 1*A*) is almost fully conserved among STIM1 and STIM2; however, STIM1/2 exhibit distinct functions, especially due to the nonconserved residue F394 in STIM1 corresponding to L485 in STIM2 (173).

Structural insights into STIM (Fig. 1*A*) have been obtained from isolated ER-luminal domains of both STIM1 and STIM2 with Ca^2+^ bound to the EF-hand via NMR (190), and a STIM1 COOH-terminal, SOAR-like, fragment (aa354–444) via X-ray resolution (29) (Fig. 1*A*). In addition, the NMR structure of a SOAR overlapping fragment (aa312–387) has been obtained in the unbound (Fig. 1*A*) as well as the Orai1 COOH terminus-bound state (161). The crystal structure of the SOAR fragment has revealed a dimeric, parallel arrangement of two monomers resulting in an overall V-shaped structure (Fig. 1*B*). The structure of each monomer resembles that of a capital letter “R,” formed by the four helices Sα1–Sα4 (180). The NMR structure of STIM1 aa312–387, including CC1α3 and CC2, displays a bend of almost 180° between these two helical domains (Fig. 1*A*). Two such fragments assemble as dimers in an antiparallel manner (Fig. 1*B*) (161). These two structures exhibit large differences, and substantial structural changes would be required to transform one form into the other.

Intra- and intermolecular interactions within and between STIM1 proteins control the activation status of STIM1 (Fig. 1*C*, see below) (44, 72, 92, 110, 180, 193). Upon store depletion STIM1 COOH terminus couples to Orai channels, thus mediating CRAC current activation (80, 111).

### Orai proteins.

**Fig. 2.**
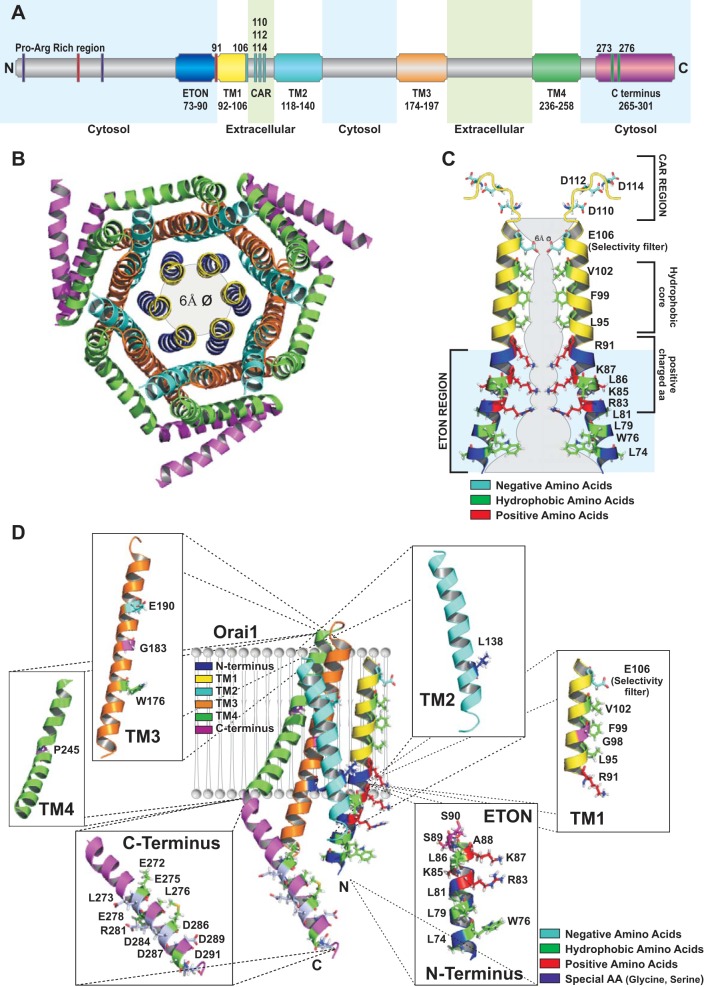
Orai1. *A*: scheme that represents full-length human Orai1 with its overall structure and residues required for Orai1 function. *B*: cartoon depicting the hexameric assembly of Orai subunits based on the X-ray crystal structure of *Drosophila* (d)Orai. Transmembrane domain (TM)1 forms the inner ring surrounding the ion-conducting pore, while the other TM domains of the 6 subunits are arranged as concentric rings around the pore. *C*: cartoon depicts the human Orai1 pore by 2 TM1 strands together with the cytosolic, helical extensions including the conserved extended transmembrane Orai1 NH_2_-terminal (ETON) region at the NH_2_-terminal side of TM1 as well as the Ca^2+^ accumulating region (CAR) region at the COOH-terminal side of TM1, as part of the loop1 region connecting TM1 and TM2. Essential regions in the pore such as the selectivity filter, the hydrophobic core, as well as residues within the ETON region are highlighted. *D*: cartoon representation of a single Orai1 subunit with the 4 TM regions and the NH_2_- as well as COOH-terminal elongated helices, depicted in distinct colors used throughout *A–D*. Moreover, all 4 TM domains, the COOH terminus, and the NH_2_ terminus are shown separately with those residues relevant to Orai1 function highlighted [amino acid (aa) numbering refers to human Orai1].

Orai proteins (Fig. 2) represent Ca^2+^-selective ion channels in the PM including three highly conserved homologs, termed Orai1-3 (15, 46, 170, 170). Each Orai monomer contains four TM segments linked via one intracellular and two extracellular loops and cytosolic NH_2_ and COOH termini (68, 105, 124) (Fig. 2*A*). The TM regions share ∼81–87% pairwise sequence identity, whereas TM1 is fully conserved among Orai proteins. The cytosolic strands, extra- and intracellular loops are less conserved, except for segments involved in direct binding of STIM1 (58). Major structural differences are found in extracellular loop3, which is much longer in Orai3, as well as in the cytosolic NH_2_ and COOH termini, which share 34% and 46% sequence homology, respectively (148). Both cytosolic strands of Orai are required for functional coupling to STIM1 (35, 80, 100, 111, 115, 121, 189). STIM1-mediated Orai currents display strongly inward-rectifying Ca^2+^ currents and a low single-channel conductance similar to what has been reported for endogenous CRAC channels (86).

For a long time it has been thought that four Orai subunits form the functional channel (31, 34, 93, 96, 105, 124); however, in 2012 the crystal structure of the dOrai channel put forward the idea that the channel may be composed of six subunits (Fig. 2*B*) (63). A closer look further reveals, besides the sixfold central axis of symmetry along the pore, an overall threefold symmetry, as dOrai monomers are arranged as three dimers within the hexameric complex (63). The ion pore, located in the center of the hexamer, is surrounded by three rings of TM domains (Fig. 2*B*). The first ring is formed by TM1 of each subunit and forms the pore, the second ring includes TM2 and TM3, and the third ring is formed by TM4 (63). Analogous to dOrai, the Orai1 proximal NH_2_ terminus likely forms an α-helical extension of TM1 of ∼20 Å (Fig. 2*C*), the so-called extended TM Orai1 NH_2_-terminal (ETON) region (35). Furthermore, TM2 and TM3 have been found to extend by a few helical turns into the cytosol (63). They are linked by a small flexible portion, loop2, that has not been resolved in the X-ray structure. Moreover, the structural resolution of the two extracellular loops is still lacking. A combination of molecular modeling and molecular dynamics (MD) simulations suggests the highest flexibility is in loop3 (51). Interestingly, the Orai COOH terminus is connected to TM4 via a highly conserved hinge region and couples to the COOH terminus of the adjacent Orai subunit in an antiparallel manner. Hence, the Orai hexamer is built up from three dimers, each with antiparallel crossing COOH termini (110, 111, 113).

## Detailed Presentation of Steps Within STIM1/Orai Signaling Cascade

### STIM1 in the resting state.

Cells in the resting state display a homogeneous distribution of STIM1 proteins, which move rapidly along the microtubules (149). In accordance with this, Baba et al. (6) have reported dynamic and constitutive movement of STIM1 before store depletion, which is governed by CC regions and the Ser/Pro-rich domain. The diffusion speed is in the range of 0.1 μm^2^/s, which is slow compared with other single-pass membrane proteins. Deletion of the cytosolic domains (STIM1 ΔC = STIM1 aa1–237) results in a twofold-increased diffusion speed comparable to other single-pass membrane proteins (28), suggesting that STIM1 proteins are slowed upon the interaction of their COOH termini.

The structure of full-length STIM1 in the resting state is still elusive. Nonetheless, the EF-SAM domains of STIM1 and STIM2 have been structurally well resolved in the Ca^2+^-bound state (190). As long as the stores are full, the EF-SAM domain forms a stable and compact monomer with a mainly α-helical structure (Fig. 1*C*) (162, 190). It is composed of a canonical and a noncanonical EF-hand that are followed by the SAM domain (158, 160, 162). Ca^2+^ is bound to the EF-hand via negatively charged aspartates and glutamates. The noncanonical EF-hand does not bind Ca^2+^ but stabilizes the monomeric state and the SAM domain (159, 190). Mechanistically, the EF-hands of two monomers form a hydrophobic cleft that binds to hydrophobic residues in the SAM domain in order to stabilize the resting state of the EF-SAM structure.

While STIM1 is inactive in the cell resting state and responds only to large drops in luminal Ca^2+^ levels, STIM2 is partially active already at smaller changes in luminal Ca^2+^ (17). This distinct behavior of the two STIM isoforms in response to cytosolic Ca^2+^ levels is mirrored in the properties and a distinct structural stability of their EF-SAM regions. The canonical EF-hand of STIM1 possesses a higher Ca^2+^ affinity than that of STIM2, while the hydrophobic and electrostatic interactions of the EF-hands and SAM are less stable in STIM1 compared with those in STIM2 (159, 190). Thus the response of STIM1 occurs in a very fast manner, but only to larger drops in ER Ca^2+^ concentrations, while STIM2 activates at smaller changes in Ca^2+^ levels, yet in a slower manner (162, 190).

The luminal EF-SAM domain is followed by a single TM domain that has just recently been reported (92) to contribute to the switch between the inactive and active states of STIM1. As shown by studies of a STIM1 gain-of-function mutant, C227W, STIM1 TM regions are supposed to cross each other with a certain angle that decreases upon store depletion. In the resting state (Fig. 1*C*), residues in the more COOH-terminal portion of the TM (aa221–232) have been shown to interact, while those in the NH_2_-terminal portion are positioned farther apart from each other (92).

Besides the luminal as well as TM domains, STIM1 cytosolic portions also control the resting state of STIM proteins. STIM1 proteins are assumed to form dimers before store depletion, since they interact with each other and isolated STIM1 fragments mostly occur as dimers (6, 64, 109, 176, 191, 193). This STIM1 dimer interaction involves the COOH-terminal regions, i.e., CC1 and CAD/SOAR. While CC1 supports dimerization, CAD/SOAR is dominant in mediating dimerization (28). Based on the crystal structure of SOAR (180), the dimeric interaction of SOAR monomers is mediated via several hydrophobic and hydrogen bond interactions (Fig. 1*B*). Specifically, NH_2_-terminal residues of one monomer (T354, L351, W350, L347) couple to COOH-terminal residues of the second monomer (R429, W430, T433, L436) (180) (Fig. 1*B*, *left*). An NMR STIM1 structure further suggests dimeric coupling via overlapping domains of CC1_α3_ (E320-A331) as well as CC2 (H355-A369) (Fig. 1*B*, *right*) (161). A cytosolic STIM1 homomerization domain (SHD) has been assigned to the segment ∼421–450, an extended portion of CC3 (109). Deletion of the SHD in a short STIM1 COOH-terminal fragment, the so-called Orai1 activating STIM1 fragment (OASF; Fig. 1*A*), results in substantially reduced homomerization (109).

The inactive, tight state of STIM1 (Fig. 1*C*) is maintained via an intramolecular clamp established via specific interactions of CC1 and CAD/SOAR. Originally, Korzeniowski et al. (72) reported an electrostatic clamp between a highly conserved acidic region in CC1 (E318/319/320/322A) and a basic region in CC2 (K382/384/385/384A). However, later reports have demonstrated that these regions are not located in close proximity (180, 193). Nevertheless, the hypothesis of an inhibitory clamp has been further supported by recent studies (110, 193), based on high Förster resonance energy transfer (FRET) or luminescence resonance energy transfer (LRET) values of STIM1 fragments labeled on both sides, which is reduced in the presence of Orai1. This intramolecular clamp is formed between CC1_α1_ and CC3 in line with Reference 92 and involves several residues as derived below.

The substitution of L248S, L251S, or L258S in CC1_α1_ and L416S or L423S in CC3 leads to a decrease of FRET as determined from a double-labeled STIM1 COOH-terminal OASF fragment, suggesting that these residues retain STIM1 in a tight, inactive conformation (110, 193). The crystal structure of *C. elegans* SOAR extended by CC1 together with functional studies has suggested that the amino acid stretch aa308–337 in CC1_α3_, which includes the residues E318/319/320/322 (72), functions as an inhibitory helix, as slightly constitutive activation of Orai1 has been observed upon deletion of aa310–337 in STIM1 (180). In addition, residues of CC2 (A369) and CC3 (L416, L423) are, because of their close proximity in the X-ray structure, supposed to be involved in intramolecular interactions (180). The R426L mutation in CC3 has been shown to promote the tight conformation of STIM1 fragments (44, 110). Moreover, Y316 in CC1_α3_ contributes to the maintenance of STIM1 in the inactive state (182). Hence, residues in both CC1_α1_ as well as CC1_α3_ and CC3 helices contribute to the inhibitory clamp for fixing the STIM1 tight, inactive state (Fig. 1*C*), while potential interaction sites on CC2 and CC3 of CAD/SOAR remain elusive so far. Recently, it has been suggested that coiled-coil interactions of L258 and L261 of CC1_α1_ with V419 and L416 of CC3 maintain the inhibitory clamp (92); however, this assumption still requires experimental validation.

## Activation of STIM1 via Signal Transduction of STIM1 NH_2_ Terminus to Its COOH Terminus

After ER store depletion, the homogeneous distribution of overexpressed fluorescence-tagged STIM1 proteins changes into punctae localized at the ER-PM junctions (84, 91, 137, 178). In line with FRET measurements, STIM1 proteins form stable oligomers (28, 83, 111), which leads to slowed movement along the microtubules (28, 85). Store-operated puncta formation is determined by both luminal as well as cytoplasmic STIM1 domains (28). Furthermore, STIM1 promotes the formation of ER-PM junctions by binding to the microtubule tip attachment protein EB1 (53). Other proteins like one of the extended synaptotagmin family, ESyt1, which interacts with phosphatidylinositol 4,5-bisphosphate (PIP_2_), and junctate, already localized at ER-PM junctions, have been reported to support the formation of ER-PM contacts (24, 52, 154, 167, 168). Additionally, an ER-resident protein, STIMATE (TMEM110), facilitates and enhances STIM1 puncta formation at ER-PM junctions because of coupling of the two proteins (69, 135). Knockdown of STIMATE results in reduced puncta formation that can be partially reversed by artificially restored junctions via Ist2, an ER-membrane protein that recruits ER to the PM (69, 135).

**Fig. 3.**
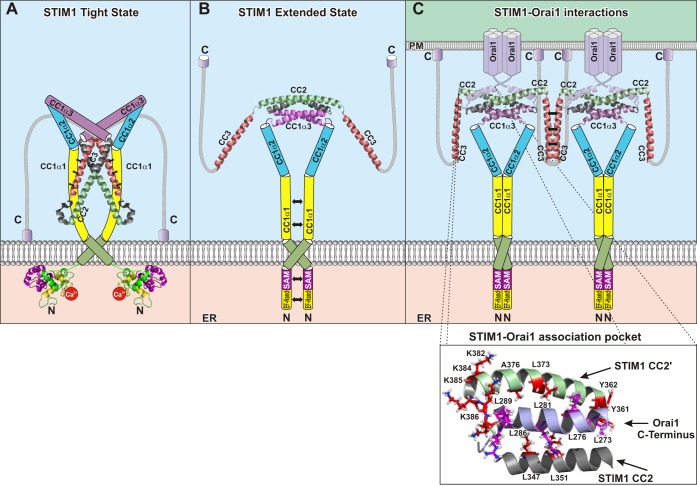
*A* and *B*: cartoons representing a hypothetical model for the coupling of STIM1 and Orai1. After store depletion, STIM1 proteins lose the Ca^2+^ bound to the luminal EF-hand and undergo a conformational change from the inactive, tight state (*A*) to the active, extended state (*B*). Thereby, the crossing angle of the TM helices alters and the inhibitory, intramolecular clamp between CC1_α1_ and CC3 is released. *C*: extension of STIM1 proteins leads to the interaction with Orai1, is accompanied by oligomerization of STIM1 proteins to larger aggregates than dimers, and involves the CC3 domains. *Inset* depicts intermolecular interactions between the STIM1 CC1α3-CC2 and Orai1 COOH terminus in the STIM1-Orai1 association pocket. PM, plasma membrane.

Loss of Ca^2+^ binding at the luminal EF-SAM domain triggers conformational changes in the NH_2_ terminus that are transmitted via the TM domain to the COOH terminus, finally culminating in the interaction with and activation of Orai channels (Fig. 3) (132). Based on an elegant study by Luik et al. (90) in which artificial luminal cross-linking of STIM1 was utilized, it is suggested that initial di- and/or oligomerization on the luminal side of STIM1 represents the first step in the activation cascade of STIM1.

Initial structural changes upon loss of Ca^2+^ at the luminal side are accompanied by an unfolding of the EF-SAM domain, based on structural and biochemical studies of the isolated STIM1 EF-SAM domain (190). Thereby the EF-SAM domain exposes hydrophobic surfaces that trigger the aggregation of STIM proteins into dimers and higher-order oligomers in solution (159, 190). In line with this, STIM1 deletion mutants lacking the whole COOH terminus have been shown to multimerize upon Ca^2+^ store depletion (28). The isolated EF-SAM domains of STIM1 and STIM2 exhibit distinct *K*_D_s for Ca^2+^ binding of ∼200 and 500 μM (190), respectively. The calculated Hill coefficient for the luminal domain of STIM1 under low ER Ca^2+^ concentrations is ∼4, which is rather high and in accordance with a potential multimeric assembly (90). Oligomerization upon loss of Ca^2+^ binding is in accordance with constitutive puncta formation of STIM1 EF-hand mutants (D76A, E87A) lacking Ca^2+^ binding as well as increased aggregation of STIM1 peptides in solution (153, 186). Further FRET studies have revealed an increase in the homomeric interaction between STIM1 proteins after store depletion that is decreased upon store refilling (83, 111). STIM1 oligomerization has been shown to occur before STIM1-Orai1 coupling (83).

Structural changes at the luminal side are supposed to trigger conformational rearrangements in the STIM1 TM domain. This assumption is based on recent studies (92) that report mutation of residues (I220, C227) in the TM region that induce constitutive activity of STIM1. These findings together with NMR and LRET studies have led the authors to suppose that store depletion decreases the crossing angle of interacting TM regions, thereby bringing the NH_2_-terminal halves (aa 214–220) closer together (Fig. 3*B*) (92). These novel findings may change the understanding of increased FRET values determined from NH_2_-terminally fluorescence-labeled STIM1 upon store depletion, which has so far been interpreted as STIM1 oligomerization. Alternatively, enhanced FRET may also result from closer proximity of luminal NH_2_ termini within a STIM1 dimer upon store depletion (132), which still requires further evaluation.

Finally, the STIM1 activating signal initiated at the luminal site is transmitted via the TM domain to STIM1 COOH terminus, resulting in further conformational changes accompanied by oligomerization (Fig. 3). In particular, the STIM1 COOH-terminal CAD/SOAR domain represents an essential determinant for STIM1 oligomerization (28). Serial COOH-terminal STIM1 truncation mutants reveal that CC1 alone is unable to stabilize an oligomeric complex, while in the presence of CAD/SOAR normal STIM1 response to store depletion is obtained (28). CC2 and CC3_ext_ within CAD/SOAR are supposed to play the dominant role in establishing the oligomeric state after store depletion, in line with the reported role of SHD (109) as well as observed puncta formation by an ER-targeted CAD/SOAR or CC3_ext_ aa388–449 domain (44). Reduced homomerization upon deletion of SHD is accompanied by abolished activation of Orai1 channels in patch-clamp recordings (109). Multiple important roles of CC3 could be further derived from the loss-of-function mutation R429C therein. This mutation allows STIM1 to transit from the closed to the open state; however, it impairs the formation of functional STIM1 higher-order oligomers and binding to Orai1 (98).

In the active state the inhibitory clamp in the STIM1 COOH-terminal portion is released, switching STIM1 from its tight, inactive state to its extended, active state (Fig. 3, *A* and *B*). FRET/LRET studies employing STIM1 fragments labeled on both sides have revealed a reduction of intramolecular FRET upon binding to Orai1 or substitution of specific residues (L248S, L251S, L258S, or the deletion of CC1) (110, 193). The release of the inhibitory clamp by these mutations is further supported by studies with ER-targeted mutated CC1 domains that lose their ability to interact with SOAR/CAD (44, 92). Furthermore, while CC1 occurs as a monomer in solution and binds to CAD, artificially cross-linked CC1 fragments form dimers that exhibit impaired interaction with CAD/SOAR (193), compatible with a di-/homomerization of the CC1 region contributing to the switch into an extended conformation (193). These results suggest that STIM1 COOH terminus adopts an extended form in the active state for exposure of CAD/SOAR and interaction with Orai1 (Fig. 3*C*). STIMATE has been reported to promote the conformational switch via coupling to CC1, thus perturbing CC1-CAD/SOAR interaction (69). Our recent study (44) additionally reveals a slight, destabilizing impact for the CC1_α2_ domain in STIM1, which is drastically enhanced by the R304W mutation therein, which fully activates STIM1 without store depletion and is associated with the Stormorken syndrome (106, 107, 114). As the CC1 region plays an important role in keeping STIM1 in the inactive state (110, 193), the disease-related R304W mutation is likely to disturb the resting, tight STIM1 conformation, promoting the switch into the extended, activated form.

A highly conserved region COOH terminal to SOAR overlapping with CC3_ext_, STIM1 (447–530), has been termed the COOH-terminal inhibitory domain (CTID; see Fig. 1*A*), as mutations within this region caused constitutive, store-independent clustering of STIM1 and activation of Orai1 (67). This region is involved in the interplay with SARAF (23), as explained in detail in *Inactivation of Orai Channels*.

The rear portion of STIM COOH terminus includes domains possessing a regulatory impact on the STIM/Orai interplay. Here, a Ser/Pro-rich region, a TRIP sequence, as well as a lysine-rich region play a significant role.

The Ser/Pro-rich region as well as the TRIP sequence (see Fig. 1*A*) located close to each other at the end of the STIM1 COOH terminus control the binding of STIM1 to microtubuli. The first includes phosphorylation sites Ser575, Ser608, and Ser621, which represent target sites for extracellular signal-regulated kinases 1/2 (ERK1/2) (126). These residues modulate STIM1-Orai signaling, as their mutation results in reduced Ca^2+^ entry as well as diminished STIM1-Orai1 coupling (126). The closely located TRIP sequence, encompassing aa642–645 in STIM1 COOH terminus (127), couples to the microtubule plus-end regulator EB1 (53, 165). Mutation of the phosphorylation sites has been also shown to regulate binding to EB1 (127), in line with reports that phosphorylation sites in the vicinity of this TRIP sequence bind to EB1 (165, 174, 195). Mechanistically, Pozo-Guisado et al. (127) have supposed that store depletion activates ERK1/2, which phosphorylates STIM1, thus triggering dissociation from EB1. This allows STIM1 multimerization and coupling to Orai1, while upon store refilling STIM1-EB1 binding avoids a prolonged active state of the STIM1/Orai1 complex.

Puncta formation of STIM proteins is further controlled by a lysine-rich region at the very end of the STIM1 protein, as truncation of the lysin-rich region impaired puncta formation while oligomerization was still preserved (83). This STIM1 COOH-terminal polybasic region represents a PIP_2_ binding domain (83). STIM2 contains an even larger lysine-rich domain and exhibits binding to PIP_2_ (41). A decrease in PIP_2_ levels has been demonstrated to affect puncta formation of STIM1-Orai1 cluster, stabilization of STIM1-PM interactions, and targeting of STIM1 to ER-PM junctions (26, 73, 172). Thus it has been hypothesized that the lysine-rich region couples ER-resident STIM1 to PM-PIP_2_, thereby enhancing the amount of STIM proteins in ER-PM junctions. Furthermore, septins that bind PIP_2_ have been shown to facilitate efficient STIM1-Orai1 communication (147).

In summary, while the inactive, tight state of STIM1 is controlled by an autoinhibitory interaction of CC1 with CAD/SOAR, the active, extended state of STIM1 is reached by release of this inhibitory clamp supported by CC1 di-/oligomerization and resulting in the exposure of CAD/SOAR for interaction with Orai1. The accumulation of STIM1 proteins in the ER-PM junctions and their oligomerization are further modulated by additional regulatory proteins.

## Coupling of STIM1 to Orai1

Activation of STIM1 leads finally to its coupling to Orai1, allowing Ca^2+^ entry into the cell. In the following the main interaction sites of the two molecular key components of the CRAC machinery are presented.

### STIM1 COOH-terminal regions.

Overexpression of the STIM1 COOH terminus (aa233–685) has been shown to be sufficient for CRAC channel activation (44, 64, 111). Furthermore, NH_2_- and COOH-terminal deletions of STIM1 COOH terminus have revealed a minimal STIM1 COOH-terminal fragment [OASF (aa233–450), CAD (aa342–448), SOAR (aa344–442), and Ccb9 (a339–444)] that is sufficient to activate Orai channels (71, 109, 121, 183). All these fragments (see Fig. 1*A*) include the CC2 (aa353–388) and CC3_ext_ (aa400–450) regions, encompassing an Orai coupling and activating domain as well as the SHD (aa421–450) (109). Although the CAD/SOAR domains of STIM1 and STIM2 share ∼80% sequence identity, they possess distinct functional properties. While CAD/SOAR of STIM1 couples strongly to Orai1 and induces robust constitutive activity, that of STIM2 has revealed only weak association with Orai1 and marginal constitutive activity. The residue F394 in STIM1 has been determined as crucial for those functional differences, as the STIM1 F394L mutant, containing the corresponding STIM2 residue, exhibits reduced coupling to Orai1 (173).

CAD has been reported to interact directly with Orai1 (121). A strong CAD interaction has been detected with the Orai1 COOH terminus, while it interacts to a weaker extent with Orai1 NH_2_ terminus, in line with Reference 35. In contrast, Orai1 loop2 has not been observed to interact with CAD (121).

A recent NMR structure (161) has drawn a first picture of how STIM1 COOH terminus potentially interacts with Orai1 COOH termini (Fig. 3*C*). Here, two STIM1 COOH-terminal fragments (aa312–387), including CC1_α3_ and CC2, bind in an antiparallel manner to two antiparallel-oriented Orai COOH termini, which fits well with their orientation in the dOrai crystal structure. Critical residues of this STIM1 fragment (Fig. 3*C*) involved in the interaction with Orai1 COOH terminus (aa272–292) to form the STIM1-Orai1 association pocket (SOAP) represent L347, L351 from the NH_2_-terminal side of one CC2 and Y362, L373, A376 from the COOH-terminal side of the other CC2′ fragment together with a cluster of positively charged amino acids, K382, K384, K385, and K386, providing electrostatic complementarity. Within this predominantly hydrophobic groove, side chains from the Orai1 COOH terminus including L273 and L276 pack against opposite faces of the SOAP (Fig. 3*C*). In line with this, mutation of L347, L351, L373, and A376 within STIM1 has already been reported to functionally disrupt the communication with Orai1 channels (48). Neutralization of the STIM1 positively charged lysine residues in the cluster has been shown to impair coupling to and activation of Orai1 (21, 72, 180). Thus the interaction of STIM1 COOH terminus with Orai1 COOH terminus is structurally well resolved and consistent with the functional effects of mutations within SOAP, while the relevant residues within STIM1 involved in coupling to the Orai1 NH_2_ terminus are poorly defined. Recently, the critical residue in the CC2-CC3 linker of the CAD/SOAR domain of STIM1, i.e., F394 (see Fig. 1*B*), determining the extent of CAD/SOAR coupling to Orai1, has been suggested to interact with exposed leucines in the ETON region of Orai1 to open the channel (173). Its mutation F394H results in loss of coupling to and activation of Orai1 (173); however, a direct binding of STIM1 to the Orai1 NH_2_ terminus in the intact channel has not yet been demonstrated. This failure of STIM1 binding may be caused by the weaker affinity to or occlusion of the Orai1 NH_2_-terminal binding site, as long as the channel resides in a state without STIM1 bound to the COOH terminus.

Two splice variants of STIM, exhibiting alterations in the COOH-terminal sequence, have been reported to alter coupling to Orai1. The alternatively spliced long variant of STIM1, STIM1L, which is most prominently expressed in muscle, includes an extra stretch of 106 residues in the STIM1 cytosolic domain after the CC3 region (30). The amino acid stretch inserted in STIM1L represents an actin-binding domain that results in permanent cluster formation, thus allowing immediate SOCE activation (30). Another study in a different cell system found STIM1L to form permanent, yet smaller, ER-PM clusters; however, Orai1 channels were not activated faster but with similar kinetics and to the maximal extent as with wild-type STIM1(141).

A STIM2 splice variant (STIM2β, STIM2.1) contains a highly conserved stretch of eight residues within the CAD/SOAR domain, resulting in inhibitory properties of this splice variant (104, 136). STIM2β by itself is unable to bind to Orai1; however, it couples to Orai1 upon heterodimerization with STIM1 or STIM2 and blocks store-operated currents (136).

### Orai1 COOH terminus.

The Orai COOH terminus forms the cytosolic extension of TM4 and is connected via a highly conserved hinge (aa261–265) to TM4 (see Fig. 2*B*) (63). Crossing COOH termini of an Orai dimer form an angle of 152° based on the dOrai crystal structure. Early colocalization and FRET studies have already revealed the Orai1 COOH terminus as indispensable for the coupling to STIM1 (80, 111, 121). Structural predictions suggest that Orai COOH termini form coiled-coil regions, known as typical structural motifs involved in homo- as well as heteromeric protein-protein interactions (97), where the probability for a coiled-coil region is 15- to 17-fold increased for Orai2 and Orai3 compared with that of Orai1 (48). In accordance, single-point mutations within Orai1 COOH terminus (L273S/D, L276S) are sufficient to disrupt the communication with STIM1 (48, 111). The structural resolution of the SOAP complex has clearly confirmed the importance of L273 and L276 as hydrophobic residues relevant for the interaction with STIM1 (161). In contrast to Orai1, double-point mutations were required in Orai2 or Orai3 COOH terminus to fully disrupt coupling to STIM1 (48). Zhang et al. (187) have reported constitutive coupling of STIM1 to Orai3, but not to Orai1, without store depletion, despite comparable activation kinetics. This constitutive coupling may depend on the strength of the Orai COOH-terminal coiled-coil region, which still needs to be examined. Besides L273 and L276, further residues R281, L286, and R289 within the Orai1 COOH terminus are involved in the interaction with STIM1 COOH-terminal SOAP (Fig. 3*C*) (161). In line with this, adjacent residues predicted not to interact with STIM1 have no effect on STIM1 activation (161).

Strikingly, the hexameric dOrai crystal structure has revealed that L273 and L276 are also involved in COOH-terminal dimerization of each Orai dimer (63). It has been shown experimentally that the hydrophobic interactions of L273/L276 are essential for channel gating, as their substitution to less hydrophobic residues results in loss of Orai1 function (117). Cysteine cross-linking of the COOH terminus pairs at those positions (L273C, L276C) impairs STIM1 binding, which could be rapidly reversed upon disulfide break (166), in line with their critical role in the Orai1 activation process.

The hinge connecting the COOH terminus to TM4 is crucial for establishing the correct conformation of Orai1 COOH termini for coupling to STIM1, as mutations therein affect STIM1 binding and CRAC activation negatively (117, 166). STIM1 binding to Orai1 hinge mutants is significantly reduced compared with wild type but only fully abolished upon additional Orai1 COOH terminus mutation (117), suggesting that the hinge brings the COOH termini in the best position required for accurate STIM1 binding.

The NMR structure of the STIM1 fragment coupled to antiparallel-oriented Orai1 COOH termini has revealed an angle of 136° in contrast to the angle of 152° of the crossing COOH termini in the dOrai crystal structure. These distinct angles may result either from different experimental procedures or from a conformational difference between dOrai and human Orai1. However, it is also likely that the angle of 136° mirrors the dimerization angle in the active state, while 152° displays the crossing angle of Orai COOH termini in the closed state. Alternatively, Hou et al. (63) have suggested a more straightened TM4-COOH terminus connection for Orai1 in the active state upon bridging of NH_2_ and COOH termini via STIM1.

### Orai1 NH_2_ terminus.

Besides the Orai1 COOH terminus, the Orai1 NH_2_ terminus also functions as a STIM1 binding partner, even if to a weaker extent than the COOH terminus (35, 121). Relevant binding residues are included in the conserved ETON region (aa73–90) forming the elongated extension of TM1 into the cytosol (see Fig. 2*C*). A truncation of the NH_2_ terminus up to aa72 leaves the channel fully active (35, 100), while upon deletion up to aa74/75 activation via STIM1 or STIM1 cytosolic fragments occurs only to a half-maximal extent and truncation up to aa76 or beyond completely abolishes Orai1 current activation (35). Specific mutations within the ETON region such as the paired L74/W76 (35), L74/L79/L81/L86 substitutions (173), or R83/K87 (35) as well as K85E (87) result in complete loss of coupling as well as activation of the Orai1 channel by STIM1.

Positively charged residues close to TM1 provide in addition to a STIM1 binding site an electrostatic barrier as well as stabilization of the elongated pore (35). Hence, almost the whole ETON region functions as binding interface for the Orai1 interaction with STIM1 and additionally provides electrostatic gating elements to fine-tune the shape of the elongated pore (138).

The ETON region is fully conserved among Orai proteins. Intriguingly, store-operated activation of Orai3 is still retained with more extensive truncations that already abolish Orai1 function. (10, 35) Thus STIM1-mediated Orai3 activation potentially involves further structures that compensate for the extensive NH_2_-terminal deletions, the location of which still remains to be resolved.

Although various residues in the Orai1 ETON region have been identified as indispensable for coupling to STIM1, a clear picture of the NH_2_-terminal binding pocket for STIM1 is still missing.

## Both Orai1 NH_2_ and COOH Termini Together Determine STIM1-Mediated Activation and Gating

The above-described resolution of STIM1/Orai1 interaction domains reveals that gating of Orai channels into the open state is established via coupling of STIM1 COOH terminus to specific residues in both Orai1 NH_2_ and COOH termini. The presence of only one functional cytosolic strand is not sufficient to allow activation via STIM1, as the deletion or mutation of either the NH_2_ or the COOH terminus leads to loss of Orai function (35, 100, 115, 117). Upon disruption of the Orai COOH-terminal binding sites, interaction with full-length STIM1 or STIM1 fragments is completely abolished (80, 111). In contrast, an impaired NH_2_-terminal binding site still allows partial coupling of STIM1 with Orai1, very likely via its COOH terminus, although functionality is lost (32, 111). These results together with biochemical binding studies of cytosolic fragments (121) suggest the Orai1 COOH terminus as the stronger binding site compared with the NH_2_ terminus. Moreover, all these results indicate that Orai1 NH_2_ terminus contributes to gating (35, 111, 138). Hence, both cytosolic strands are indispensable for Orai activation; however, the question remains of how STIM1 attaches to the binding sites of the Orai channels, enabling their activation. One might hypothesize that STIM1 couples initially to the COOH terminus, because of their stronger binding affinity, and subsequently to the NH_2_ terminus. Potentially, activation of Orai1 channels occurs via a bridging of Orai1 NH_2_ as well as COOH terminus that is most likely accomplished by the CAD/SOAR domain. It may be further assumed that STIM1 binding to the Orai COOH terminus, in addition to a simple anchoring, also involves rearrangements in the TM4 extensions that facilitates concomitant interaction of CAD/SOAR with Orai1 NH_2_ terminus.

Recent studies (100, 115, 117) have examined the requirement of STIM1 interaction with both Orai1 NH_2_ and COOH termini, utilizing local enrichment of STIM1 COOH terminus by tethering CAD fragments to either the NH_2_ or the COOH terminus. The deletion mutants Orai1-Δ1–76 and Orai1-Δ276–301, which are nonfunctional upon stimulation with STIM1, remain partially active in the presence of tethered CAD (117). Nevertheless, a minimal portion of Orai1 NH_2_ terminus (aa77–90) and COOH terminus (aa267–275) is still required also for preserved activation via tethered STIM1, as evident from loss of function of Orai1-Δ1–77 or Orai1-Δ273–301 tethered to CAD/SOAR. Single-point mutations either in NH_2_ or COOH terminus impairing function upon stimulation via cytosolic STIM1 fragments do not inhibit activation of analog Orai1 mutants with tethered STIM1 fragments (115). Thus the local attachment of CAD/SOAR here apparently circumvents the requirement of two functional cytosolic strands as usually seen with full-length STIM1 or cytosolic STIM1 fragments, probably by overcoming decreases of affinity with local tethering. Only the introduction of disturbing mutations in both NH_2_ and COOH termini (W76C, K85E, L273S, L276D) results in loss of function of the CAD/SOAR-Orai linked constructs. These studies underline that Orai1 NH_2_ and COOH termini contribute synergistically to the interaction with STIM1 for control of gating or ion selectivity. Palty and Isacoff (115) have suggested, alternative to a stepwise STIM1 coupling, which initially starts with STIM1 binding to the Orai1 COOH terminus and subsequently to the NH_2_ terminus, that the NH_2_- and COOH-terminal binding sites assemble to a distinct binding pocket for STIM1 that controls gating and selectivity of Orai channels.

The NMR structural resolution of a complex of a STIM1 COOH-terminal fragment and Orai1 COOH terminus complemented by mutagenesis studies has already characterized the binding pocket of STIM1 (SOAP) embedding the Orai1 COOH terminus (see Fig. 3*C*). It is of note that this complex represents only part of the interactions involved in physiological, full-length Orai1 activation, as particularly the Orai1 NH_2_ terminus and the CC3_ext_ domain of STIM1 are missing. Here, complexes of larger STIM1 COOH-terminal portions together with additional Orai1 cytosolic strands or the full-length Orai1 may ultimately provide structural resolution closest to the physiological STIM-Orai1 interaction. Moreover, although coimmunoprecipitation studies have revealed no interaction with the third cytosolic Orai domain, i.e., the loop2 linking TM2 and TM3, its role in the cooperative coupling of STIM1 to Orai1 NH_2_ and COOH termini still needs to be reevaluated.

Furthermore, a clear picture of the structural requirements as well as molecular determinants of STIM1 for its coupling to Orai1 is still missing, as, strikingly, the CAD/SOAR crystal structure and the STIM1 fragment NMR structure display quite distinct orientations (Fig. 1*B*, Fig. 3, *A* and *B*). A switch between these two structures would require substantial conformational changes, conceivable with a scissorlike opening at the pivot (around aaY361) and separation of the CC2 domains to form the SOAP. While the STIM1 fragment NMR structure (161) adopts a conformation that fits well with an interaction with the antiparallel-oriented COOH termini in the dOrai crystal structure, how the SOAR crystal structure in its present form would couple to Orai1 remains unknown.

## Stoichiometry of STIM1/Orai1 Complex Required for Its Activation

The coupling of STIM1 to Orai1 upon store depletion results in the formation of an oligomeric, heteromeric complex, where the exact stoichiometry of interacting subunits still remains unclear. Several studies have reported that the activation of Orai1 depends on the number of interacting STIM1 proteins (59, 79, 145). Scrimgeour et al. (145, 146) have demonstrated that the extent of CRAC current inactivation depends on the number of STIM1 molecules that bind to the Orai1 channel. The fewer STIM1 proteins interact with Orai1, the fewer CRAC channel currents inactivate. Furthermore, an enhanced STIM1-to-Orai1 ratio increases selectivity for divalent cations as well as decreasing 2-aminoethoxydiphenyl borate (2-APB)-mediated current potentiation (145, 146).

Patch-clamp studies with cells containing varying STIM1-to-Orai1 concentration or fusion proteins of Orai1 (59) and tandem dimers of extended CAD have shown that eight STIM1 molecules (79) are needed for maximal CRAC current activation and inactivation. Nevertheless, only one or two STIM1 proteins are sufficient for coupling to Orai1 channels at the ER-PM junctions (59). Utilization of Orai1 and Orai1 V102A constructs tethered to single and tandem CAD/SOAR domains has revealed an enhancement of Ca^2+^ selectivity upon an increased amount of bound STIM1 fragments (101). Thus these studies suggest that activation of CRAC channels is not established in an “all-or-none” manner but evolves as a graded process involving up to eight STIM1 molecules (79). However, from the point of view of the crystallized hexameric Orai structure, how eight STIM1 molecules may couple to six Orai subunits is elusive. The recent structural NMR resolution of the SOAP structure has revealed a coupling of a STIM1 dimeric fragment to two Orai COOH termini, suggesting rather a STIM1-to-Orai1 ratio of 1:1 in an active complex (161). Thus six STIM1 molecules potentially attach to the six Orai proteins, or in other words one STIM1 dimer interacts with each of the three Orai dimers. Alternatively, to fulfil the requirements of a STIM1-to-Orai1 ratio of 2:1, 12 STIM1 proteins are needed to activate an Orai hexamer. A study by Zhou et al. (194) has hypothesized a completely new STIM1-Orai1 coupling model utilizing the F394H mutation (173) that disrupts STIM1 interaction with and activation of Orai1. SOAR-tandem constructs, containing either two wild-type SOARs or one wild-type together with one mutated F394H SOAR, activate Orai1 to comparable current levels and display similar interaction as determined by FRET. Only a SOAR tandem with both monomers carrying the F394H mutation results in loss of coupling to and activation of Orai1 channels. Furthermore, an expressed PM-fixed Orai1-COOH terminus construct yields half-reduced interaction with the SOAR tandem carrying one F394H mutation compared with the SOAR wild-type tandem. On the basis of these results, the authors suggest a unimolecular interaction between one Orai channel subunit and one STIM1 within the STIM1 dimer. Furthermore, they hypothesize that the other STIM1 within the STIM1 dimer probably couples to an Orai subunit of an adjacent Orai1 hexamer, thus providing the ability for Orai1 channels to arrange into clusters. It is of note that this interesting model is based on the assumption that STIM1 (or STIM1 F394H mutant) interaction with the PM-targeted Orai1 COOH terminus mimics those occurring in the full-length Orai1 channel complex, where the COOH termini within an Orai1 dimer are arranged in an antiparallel dimeric form, at least as found in the crystallized dOrai channel structure. Mechanistically, the proposed unimolecular manner of STIM1-Orai interactions leaves open the question of how one STIM1 fragment is able to induce alterations within an Orai1 COOH terminus dimer finally resulting in channel activation. Potentially, to fill both COOH-terminal STIM1 binding sites of one Orai dimer, two STIM1 monomers, each of another STIM1 dimer, might interact with the COOH termini to induce activation. Further evidence may be obtainable by construction of a STIM1 monomeric form provided that it can still attain a conformation for interaction with and activation of Orai1 channels. A clustering of Orai channels is, however, also conceivable via a bimolecular binding of a STIM1 dimer to an Orai1 dimer, as the STIM1 proteins possess the ability to form clusters in the activated state involving the CC3_ext_ domain (44). Hence, it is tempting to speculate that the CC3_ext_ domains may not only contribute to the formation of oligomeric STIM1 assemblies of the Orai1 channel complex they are interacting with (161) but additionally enable the linkage of adjacent Orai channels into larger clusters. However, further experimental evidence is required for whether and how STIM1 dimers may arrange Orai channels into clusters.

## Gating of Orai Channels

### Permeability of CRAC/Orai channels.

CRAC channels exhibit an exquisite Ca^2+^ selectivity with a 1,000 times higher permeation for Ca^2+^ than for Na^+^ (61). Thus Orai Ca^2+^ currents display strong inward rectification with a reversal potential higher than +60 mV (62, 86). CRAC channels have a very low single-channel conductance that has so far precluded direct recording of single-channel currents. Recently, however, optical recordings have been obtained with an approach in which a genetically encoded calcium indicator has been fused to Orai1 proteins (39). Multiple conductance states of the open channel together with periodic fluctuations in Orai1 activity have been observed in presence of CAD/SOAR (39). Orai channels also conduct small monovalent ions such as Na^+^, Li^+^, or K^+^, but only as long as the monovalent solution is free of divalent ions. The blockage of monovalent Orai currents occurs by addition of Ca^2+^ in the micromolar range (8, 61, 76, 130, 131, 164). A single Ca^2+^ ion is supposed to inhibit the large monovalent Orai currents, and Ca^2+^ block occurs in a voltage-dependent manner (130, 179). Similarly, L-type Ca_V_ channels also bind Ca^2+^ tightly to a high-affinity binding site to abolish Na^+^ entry (140). However, in contrast to L-type or TRPV6 channels, Cs^+^ is impermeant for Orai channels. The reason for that has been supposed to lie in a very narrow pore diameter of 3.8–3.9 Å, which is significantly smaller than that of Ca_V_ channels (130, 179). Interestingly, resolution of the dOrai crystal structure revealed a diameter of 6 Å at the narrowest region of the pore (Fig. 3*B*), which is obviously at variance with the diameter obtained experimentally by Yamashita et al. (179). Potentially the pore diameter is altered by the interaction of Orai with STIM1.

### Ion conduction pathway of the Orai pore.

The ion conduction pathway of multimeric Orai channels is formed by TM1 together with the ETON region comprising residues 74–90 (see Fig. 2*C*). A first picture of the pore has been drawn based on coherent electrophysiological results, cysteine scanning mutagenesis studies, and determination of the block of Orai currents by either oxidative cross-linking of cysteines or cadmium (99, 179, 192). Finally, this perception of the Orai pore has been confirmed by the later published dOrai crystal structure (63), which revealed an association of six TM1 helices in the middle of the Orai complex constituting the conduction pathway. The Orai pore (see Fig. 2*C*) consists of an external vestibule, the selectivity filter, and a hydrophobic cavity, followed by a basic region (63). Briefly, the external vestibule includes negatively charged residues that attract Ca^2+^ ions (51). The selectivity filter is exclusively formed by E106 at the more extracellular side of TM1, which is followed by V102, F99, and L95 constituting the hydrophobic cavity. Finally, at the more cytosolic side the pore ends with a basic region including R91, K87, and R83 (32, 63, 138).

**Fig. 4.**
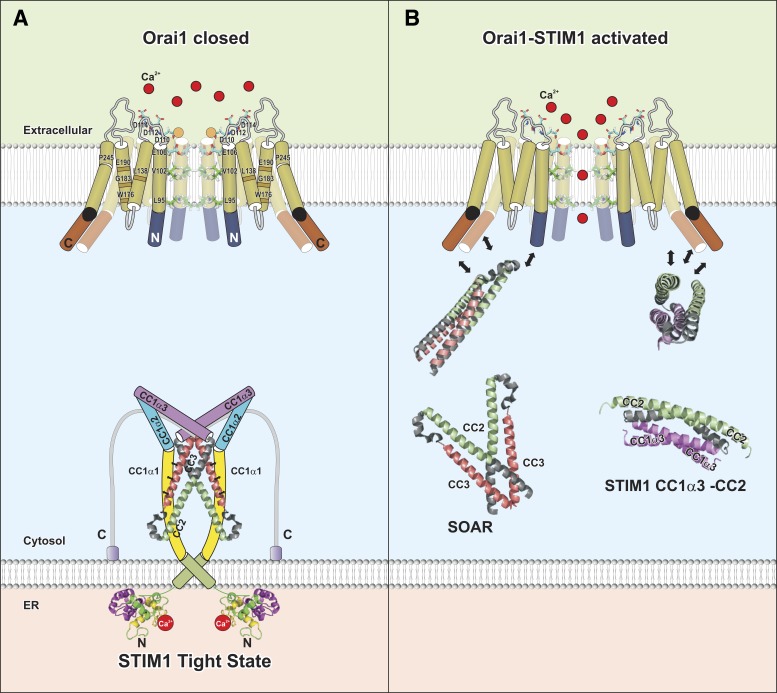
Hypothetical model for gating of Orai1 channels: cartoon representation of the closed (*A*) and open (*B*) states of Orai1 when coupled to STIM1-CAD/SOAR or STIM1-CC1_α3_-CC2. *A*: in the closed conformation of the Orai1 channel, TM1 is locked in a place that prevents ion permeation. The conformation of TM1 is adjusted by TM2, TM3, and TM4, potentially by the highlighted residues, which can induce constitutive activity upon their mutation. STIM1 assumes the tight, inactive state. *B*: coupling of Orai1 to STIM1 (here the 2 structurally resolved STIM1 fragments are displayed) induces the open state of Orai1. Both Orai1 NH_2_-terminal and COOH-terminal interactions with STIM1 are required, to alter the angle at P245 in TM4 and induce TM1 reorientations that switch the hydrophobic gate(s) and render the Orai1 pore conducting. Further alterations in the orientation of the other TM regions are likely to contribute. Focus in this panel is not laid on stoichiometry and how STIM1 couples, but conformational changes occurring upon Orai1 gating are emphasized.

The external vestibule (see Fig. 2*C*) is formed by the TM1-TM2 loops, each of which includes three negatively charged residues (D110, D112, D114). Their triple mutation to alanines alters ion selectivity and induces a widening of the pore (179), while single cysteine or alanine substitutions leave the Orai1 mutants' Ca^2+^ selectivity unaltered (51, 99). These loop segments have been shown via the substituted cysteine accessibility method (SCAM) to tightly couple with large (>8 Å) as well as small (<3 Å) positively as well as negatively charged MTS reagents (99). These results suggest that the first loops surround a large vestibule that is able to accommodate bulky compounds of different size and charge. Furthermore, disulfide cross-linking studies as well as Cd^+^ block experiments have suggested that these loop regions are very flexible (102). Recent data by Frischauf et al. (51) have revealed that these negatively charged residues (D110, D112, D114) function as a Ca^2+^ accumulating region (CAR) of Orai channels (Fig. 4), probably decreasing the energetic barrier for Ca^2+^ ions to enter the pore. In particular, D110 and D112 have been demonstrated by MD simulations to function as transient Ca^2+^ binding sites before Ca^2+^ ions reach the selectivity filter located 1.2 nm away from CAR. A substitution of D110 to an alanine results in reduced permeation of Ca^2+^, particularly at lower extracellular Ca^2+^ concentrations, likely because of a shift of Ca^2+^ binding to D112 and D114 and thus an enhanced energy barrier between CAR and the selectivity filter. Besides the interaction of the TM1-TM2 loop1 with Ca^2+^, additional electrostatic interactions have been discovered between TM1-TM2 loop1 and the TM3-TM4 connecting loop3, enabling fine-tuning of Ca^2+^ accumulation to the pore. Cysteine-induced cross-linking of D112 and R210 results in reduced store-operated current densities, which were enhanced by 400% upon breakage of disulfide bonds. Hence, the loop3 apparently competes with Ca^2+^ binding to loop1, thereby finely adjusting the Ca^2+^ permeation (51).

Interestingly, at analogous sites where Orai1 contains the three glutamines, Orai2 and Orai3 contain a mixture of glutamates, glutamines, and aspartates. While homomeric Orai channels exhibit inward-rectifying, Ca^2+^-selective currents, heteromeric assemblies of Orai isoforms, which display an asymmetric composition of the glutamates and aspartates, are less Ca^2+^ selective with increased Cs^+^ permeation (142). These results further support the concept that this acidic Ca^2+^ coordination site in the first loop besides Ca^2+^ permeation also regulates Ca^2+^ selectivity of Orai channels.

Upon the attraction of Ca^2+^ ions via those negatively charged residues in the loop1 region they are further guided to the selectivity filter, solely formed by E106 located at the extracellular end of TM1 (128, 169, 181). Based on the hexameric crystal structure (see Fig. 3, *B* and *C*), the selectivity filter is composed of six glutamate residues (63). Electrophysiological studies have reported that Orai1 E106A/Q lost function, while E106D reduced the Ca^2+^ selectivity and widened the pore diameter to ∼5.4 Å (179). This enhanced pore size is accompanied by a further relieved steric hindrance for Cs^+^, thus leading to diminished Ca^2+^ selectivity (179). Cysteine cross-linking has revealed close proximity of E106 residues and higher rigidity of TM1 helix residues aa99–104 along the Orai pore (192). Thus close positioning of E106 residues in the hexameric pore as well as rigidity of the adjacent helical stretch have been supposed to coordinate Ca^2+^ at E106 (192). In line with, recent MD simulations have revealed under equilibrium conditions one Ca^2+^ ion bound to E106 (51). This ring of negatively charged residues provides a negative electrostatic potential forming the selectivity filter and potentially functions as a gate for Ca^2+^ ions to enter the channel (101).

Deeper in the Orai1 pore, the selectivity filter is followed by the wide, hydrophobic cavity including V102, F99, and L95 (see Fig. 2*C*). These residues, except F99, point directly into the pore, which is in line with their ability to dimerize upon cysteine substitutions and suggests that they provide extensive van der Waals forces to each other (192). Moreover Cd^+^ blockage studies reveal effective inhibition of V102C, G98C, and L95C mutants (102). Since Orai1 V102C becomes constitutively active in the absence of STIM1, somehow locking Orai1 into an open conformation, V102 has been suggested to form the hydrophobic gate (Fig. 4) (101). In line with this, MD simulations for dOrai channel have revealed that a V174A mutation (corresponding to V102A in human Orai1) provides a more favorable permeation pathway than the wild-type channel based on potentials of mean force for the translocation of a single Na^+^ ion (37). However, the simulations have been performed in the presence of Na^+^ but not Ca^2+^.

V102 has been further established as the hydrophobic gate because STIM1 coupling to Orai1 induces conformational changes close to E106 and V102 that have been observed via alterations in the luminescence of Tb^3+^ bound in the pore (54). Hence, Orai gating and ion selectivity are closely coupled, since upon channel activation V102 and E106 together undergo a conformational change and are located only one helix turn apart from each other (54, 101).

Additionally, the hydrophobic cavity includes G98 (see Fig. 2, *C* and *D*), suggested to function as a flexible gating hinge (184), common for diverse other ion channels (122, 175, 188). It is assumed that G98 provides flexibility to the upstream pore-lining region, enabling Ca^2+^ ions to easily pass the pore after the selectivity filter. The Orai1 G98D mutant exhibits nonselective, constitutively active currents. As the additional introduction of the permeation-blocking R91W mutation is ineffective in inhibiting the activity of Orai1 G98D in contrast to Orai1 V102A/C channels, one may assume a larger and/or less flexible widening of the pore with the former G98D mutation.

The hydrophobic portion of the pore is followed by a basic region extending into the cytosol, forming the elongated pore of Orai1. This extended region, the so-called ETON region (35), likely provides an electrostatic barrier via the three positively charged amino acids R91, K87, and R83 (see Fig. 2*C*). Based on the crystal structure, all three residues point into the pore, which is in line with R91C exhibiting cysteine cross-linking (102, 192). In the closed-channel state, these residues are assumed to block Ca^2+^ entry probably either due to bound anions or simply by electrostatic repulsion in this region (63). Single-point mutation of R91 to a hydrophobic residue that is associated with SCID assumedly generates a robust hydrophobic barrier (63) that hinders Ca^2+^ flow through the elongated pore. The ETON region not only functions as a potential gate but also contributes a binding interface for STIM1 (35).

In aggregate, permeation through Orai channels is initiated via the attraction of Ca^2+^ ions by the Ca^2+^ accumulating region in loop1, and then Ca^2+^ ions are guided to the selectivity filter constituted by the single glutamate E106 of each Orai1 subunit. Afterwards, Ca^2+^ ions pass the hydrophobic regions and are finally released into the cytosol, potentially via the repulsion of positive-charged residues at the end of the pore.

## TM Regions Surrounding TM1 Further Contribute to Orai Gating

Gating of Orai channels into the open state occurs via STIM1 binding to both Orai1 COOH and NH_2_ termini (Fig. 4), thereby inducing a conformational change within the Orai hexamer. The TM1 helices lining the permeation pathway for Ca^2+^ in Orai channels are probably opened via a change in the orientation of pore-lining residues of TM1, allowing Ca^2+^ to pass the pore. Which structural change actually occurs in the Orai1 channel complex and particularly within the TM1 helices remains unknown so far.

Mutagenesis studies have revealed specific residues that are required to keep Orai channels in the closed state. As mentioned above, Orai1 V102A/C and Orai1 G98D yield nonselective, constitutively active currents. These results suggest that V102 and G98 (see Fig. 2*D*, Fig. 4*A*) are involved in keeping Orai1 channels in the closed state (101, 184).

Besides residues in TM1, it is known that several residues in TM2, TM3, as well as TM4 also affect gating or contribute to the control of the closed state of the pore even if they do not line the pore, as their mutation leads to altered gating properties or even constitutively active channels.

In TM2, L138 has been suggested to keep Orai1 channels in the closed state, as its mutation to a phenylalanine (L138F) leads to constitutively active currents (40). This residue is located at the TM1-TM2 interface (Fig. 2*D*, Fig. 4*A*). The L138F mutation has been found to cause tubular aggregate myopathy (40).

In TM3, mutation of several residues has been shown to modulate selectivity and gating of Orai1 channels (Fig. 2*D*, Fig. 4*A*). Mutation of the tryptophan to a cysteine W176C induces constitutive, but less Ca^2+^-selective, currents (157). The glycine G183 in TM3 is required for unconfined gating of Orai1 channels, as its substitution to an alanine fully abolishes store-operated activation but leads to altered sensitivity to 2-APB (157). The analog Orai3 G158C exhibits altered kinetics in response to 2-APB and an impaired closing of the channel upon 2-APB washout (4). These effects of G158C have been attributed to a potential TM2-TM3 interaction with an endogenous cysteine, C101, which probably controls the activation state of Orai1 channels (4).

Additionally, the conserved glutamate E190 in TM3 (Fig. 2*D*, Fig. 4*A*) is involved in the maintenance of Orai's Ca^2+^ selectivity. Its substitution to an alanine or glutamine results in increased permeation of Cs^+^, a pore diameter increased to 7 Å, and decreased 2-APB activation (179). It has been supposed that E190 allosterically affects the pore, probably via alterations of intramolecular TM interactions.

Furthermore, P245 located at the kink of TM4 (Fig. 2*D*, Fig. 4*A*) is involved in controlling the closed state of Orai1 channels. The Stormorken disease-related mutation P245L (114) has been shown to cause constitutive activation, although the residue is located far away from the pore. Interestingly, substitution of this proline by all the other amino acids leads to constitutive activation, suggesting that only the proline keeps Orai1 channels in the closed state (115).

In summary, not only residues in TM1 but also several others in the surrounding pore TM regions are involved in keeping Orai1 channels in the closed state. Strikingly, G98, G183, and P245 are all situated in the same membrane plane (117). Hence, it seems that even if these residues are not lining the pore they somehow contribute indirectly to gating and/or permeation of Orai channels. Constitutively active mutants probably mimic, at least partially, one step of the conformational change occurring upon STIM1 binding. It may be hypothesized that the simultaneous binding of STIM1 to both the NH_2_ and COOH termini of Orai1 initiates a synchronized rearrangement of TM helices leading to the open state (Fig. 4*B*). Here STIM1 binding to Orai1 may alter the orientation of the NH_2_ and COOH termini, which presumably affects TM helix arrangements, partially destabilizes the packed closed conformation, and gates the channel finally via TM1 reorientation into the open state (Fig. 4*B*).

## STIM1 Tunes Orai Channel Gating Characteristics

Interestingly, STIM1 is not only responsible for CRAC channel gating but also tunes the hallmarks of CRAC channels, i.e., high Ca^2+^ selectivity and fast Ca^2+^-dependent inactivation. Functional coupling of STIM1 to Orai1 induces gating but also regulates selectivity. This has been initially revealed by the Orai1 V102A/C mutants. Its constitutive, nonselective currents regain Ca^2+^ selectivity in the presence of STIM1. Thus STIM1 is able to fine-tune the ion selectivity of the constitutively open Orai1 V102A/C channels. High Ca^2+^ selectivity is only established as long as STIM1 binding is intact to both the NH_2_ as well as COOH terminus of Orai1 V102A/C (35, 100, 101). In contrast to Orai1 V102A, Orai1 G98D cannot regain Ca^2+^ selectivity in the presence of STIM1 (184). In the absence of STIM1, Orai1 V102A/C displays poor Ca^2+^ selectivity and enables permeation of Na^+^, Cs^+^, and several other large cations, which are not permeant through wild-type Orai1/CRAC channels (101). The coupling of Orai1 V102A/C to STIM1 enhances Ca^2+^ selectivity together with a narrowing of the pore, thus restoring dimensions of the wild-type Orai1 channel.

Similarly, constitutively active Orai1 P245L currents display reduced selectivity compared with that obtained by store depletion in the presence of STIM1. Furthermore, Orai1 P245L exhibits fast Ca^2+^-dependent inactivation (CDI) in the presence of STIM1 but not, however, in its absence (117). Hence, it seems that despite the fact that the P245L mutation induces a constitutively active Orai1 state, STIM1 is indispensable to fully attain the selectivity as well as fast CDI of wild-type STIM1-activated Orai1 currents.

It is not unique to the V102A/C and P245L mutant channels that STIM1 tunes their ion selectivity. Wild-type Orai1 currents have also been shown to display increasing ion selectivity with progressive amounts of bound STIM1 (101). Hence, both mutants potentially exhibit an intermediate active state, which reaches all of the CRAC channel characteristics only in the presence of STIM1. How STIM1 binding causes the changes in ion selectivity or inactivation remains to be elucidated at a structural level. Nevertheless, one might hypothesize that STIM1 binding to the NH_2_ terminus connected to TM1 influences the conformations of the selectivity filter. Further STIM1 coupling to the Orai COOH terminus is likely linked to structural alterations in the TM4 domains, which propagate further via TM3 and TM2 to TM1 regions (Fig. 4*B*).

## Inactivation of Orai Channels

Orai/CRAC channel activity is inhibited via so-called Ca^2+^ dependent inactivation (CDI), which represents an important feedback mechanism to control intracellular Ca^2+^ concentrations. Ca^2+^-dependent inactivation of Orai channels is separated in fast (FCDI) and slow (SCDI) CDI (23). FCDI has been reported to occur within milliseconds after channel activation (120, 129) and is typically observed during a hyperpolarizing voltage step as a decrease in CRAC currents over tens of milliseconds (61, 197). In contrast, SCDI takes several minutes for completion (120, 129).

CDI is controlled by several components in the CRAC channel signaling cascade: STIM1 and Orai as well as additional regulatory proteins, calmodulin (CaM) and SARAF. Several cytosolic regions of the STIM1 COOH terminus and Orai have been identified to determine Orai channel inactivation.

STIM1 has been suggested to define the extent of inactivation in dependence of the STIM1-to-Orai1 ratio (145, 146). Furthermore, an acidic cluster (aa475–483) termed the CRAC modulatory domain (CMD) in STIM1 COOH terminus is indispensable for FCDI of Orai/CRAC channels (33, 75, 112). Alanine substitutions of these negatively charged amino acids in the CMD reduce inactivation of all Orai1-3 channels as well as native CRAC currents in rat basophilic leukemia (RBL) cells (33, 75). Moreover, it has been shown for Orai1 V102A as well as Orai1 P245L that, despite their constitutive activity, STIM1 is required to induce inactivation characteristics typical for STIM1-mediated Orai1 currents.

FCDI and SCDI of STIM1-Orai1-mediated currents have been recently reported to be controlled by an additional ER-resident protein, SARAF (23, 67, 116). While the mechanism for FCDI regulation via this auxiliary protein is unclear, the role of SARAF in SCDI has been uncovered (23, 67, 94). SARAF interacts with the CAD/SOAR domain of STIM1, however, only in the presence of Orai1 (67). SARAF is regulated by a STIM1 domain downstream of SOAR, the so-called COOH-terminal inhibitory domain (CTID), which underlies a complex mechanism of interaction of SARAF with different portions of CTID (67). In the presence of SARAF, STIM1-Orai1-mediated currents display SCDI, which is reduced when the polybasic cluster is lacking, PIP_2_ is depleted, or STIM1 proteins are targeted to PIP_2_-poor regions (94). The coregulation of STIM1 and SARAF is further supported by septin 4 and ESyt1, which contribute to keep STIM1/Orai1 complexes in PIP_2_-rich regions (23, 94).

All three Orai channels exhibit FCDI within the first 100 ms of a voltage step; however, it is three times stronger for Orai3 compared with Orai1 or Orai2 (75, 86, 142). While Orai1 currents display a late reactivation phase, Orai2 and Orai3 channels further exhibit a slow inactivation phase over a 2-s voltage step (86, 142). In contrast to overexpressed Orai proteins, the inactivation of native CRAC currents in RBL mast cells (33, 197) is much more distinctive and lacks the characteristic reactivation phase seen with Orai1, suggesting that further players account for the inactivation of native CRAC channels.

Located within the Orai1 NH_2_ terminus, the specific proline/arginine-rich region has been shown to mediate reactivation (49). Moreover, FCDI has been shown to be regulated by CaM binding to the Orai NH_2_ terminus. Studies with isolated Orai1 and Orai3 NH_2_-terminal fragments have revealed binding to CaM. In accordance, mutations within Orai1 NH_2_ terminus (A73E; W76E/A/S, Y80E) disrupting as well as deletions in the Orai3 NT removing the CaM binding site result in loss of FCDI (10, 49, 112). In line with this, with Orai1 NH_2_ terminus mutagenesis studies (112) a crystal structure of Orai1 NH_2_ terminus and CaM in complex has confirmed an interaction of CaM with Orai1 W76 and Y80 (89). Nevertheless, overexpression of CaM-EF-hand mutants together with STIM1 and Orai1 does not result in reduced inactivation (88), suggesting that CaM is not prebound but rather associates with the Orai1 channels after increases in cytosolic Ca^2+^ concentrations. Furthermore, as W76 and Y80 are facing the interior of the pore in the dOrai1 crystal structure, it remains unclear how CaM fits into this area without strong conformational changes into the open state (132).

As demonstrated by chimeric and mutational approaches, the intracellular loop of Orai1 connecting TM2 and TM3 modulates fast and slow inactivation upon a hyperpolarizing voltage pulse (49, 156). Substitution of the amino acid stretch 151-VSNV-154 by four alanines impairs fast inactivation. Furthermore, overexpression or intracellular perfusion of a short peptide encompassing this region reduces CRAC currents. Hence, loop2 might either function as blocking particle or act in an allosteric manner on inactivation.

Orai COOH terminus exhibits a complex role in the modulation of fast inactivation. Fast inactivation is diminished for Orai2 and Orai3 chimeras with the COOH terminus substituted by that of Orai1 (75), which has been attributed to three glutamates only present in Orai2 and Orai3 COOH termini (75). In contrast, the swap of Orai1/3 COOH termini leaves fast inactivation unaffected. It is of note that the cytosolic NH_2_ and COOH termini and loop2 affect Orai inactivation/gating in a cooperative manner (49). Additionally, fast inactivation is determined by negatively charged residues within the outer pore vestibule of Orai1 channels (179). Hence, Orai channels employ diverse regions to control CDI. The interpretation of potential inactivation sites may require a strict control of STIM/Orai stoichiometry (59, 145).

In summary, FCDI of Orai channels is regulated via negatively charged residues in both STIM1 and Orai. Moreover, the second intracellular loop as well as CaM acting on Orai NH_2_ terminus affects fast inactivation. SCDI of Orai currents is regulated via STIM1 and SARAF; however, whether SCDI also involves Orai domains remains so far unclear.

## Perspectives

Within the past 10 years substantial advances have already been made toward the identification of the molecular components of the CRAC channel family together with access to their three-dimensional atomic structures. Nevertheless, several questions are still unresolved.

Regarding STIM1, only a portion of its cytosolic COOH terminus has been characterized at atomic resolution, where currently the whole and a part of the CAD/SOAR domain are available, showing significant differences in their structure. Here, further structural studies are required, particularly with longer STIM1 COOH-terminal fragments for clarification of the intra- and intermolecular interactions that contribute to the regulation, switching STIM1 from its tight, inactive to its extended, active conformation. Locking the extended conformation by L251S or L258G mutation may help to obtain structures that mimic the active state of cytosolic STIM1. Moreover, the crystal structure of Orai1 displays the closed state, while the structure of the open state is still unresolved. Here, constitutively, active Orai channels (e.g., Orai1 P245X) that are locked in an open state may help to address open channel conformation(s) by X-ray crystallography. The detailed molecular mechanism for how Orai channels are gated via STIM1 into the open state is only partially understood. So far, the NMR structure from a complex of a STIM1 fragment and the Orai1 COOH terminus has led to the characterization of the association pocket (SOAP), yet that with the Orai1 NH_2_ terminus is still missing. Moreover, the interaction of STIM1 with the full-length Orai1 channel will require structure-resolving studies of these proteins in complex. These studies may reveal the conformational changes that take place upon Orai channel activation including rearrangements of the cytosolic strands linked with those among the TM regions. Single-particle cryo-EM (7, 25) may provide an additional, valuable approach here, as it has been quite successfully used with ion channels such TRPV1 (81) or the IP_3_ receptor (45), obtaining almost similar resolutions as with crystallography.

The current achievements in understanding of the communication between STIM and Orai proteins will further enable us to widen our knowledge on physiological downstream signaling of the CRAC channels (70, 118). Sophisticated control of this communication by light (55, 65) has recently opened the Ca^2+^ signaling field to optogenetics. Potential proteins modulating the STIM/Orai communication, such as CRACR2A (155), SARAF (23, 116), Septin (147), or STIMATE (69), add further complexity to the native CRAC channel system and have the potential for a fine-tuned interference with drugs. Although several CRAC channel inhibitors are already available (66, 119, 133), their precise site of action has so far remained unclear. Besides the classical pore blockers, the complex machinery behind the CRAC current assumedly offers numerous interventions to interfere with protein-protein interactions in the CRAC channel complex to discover novel CRAC channel modulators with significant therapeutic potential in immune deficiency, autoimmune, or allergic disorders.

## GRANTS

This work was supported by the Austrian Science Fund (FWF projects P25210 and P27641 to I. Derler and FWF projects P25172 and P27263 to C. Romanin). I. Jardin was supported by MINECO (Grant BFU2013-45564-C2-1-P) and Junta de Extremadura-FEDER (GR15029).

## DISCLOSURES

No conflicts of interest, financial or otherwise, are declared by the author(s).

## AUTHOR CONTRIBUTIONS

Author contributions: I.D. and I.J. prepared figures; I.D. and C.R. drafted manuscript; I.D., I.J., and C.R. approved final version of manuscript.
